# Nutrigenomics and Beef Quality: A Review about Lipogenesis

**DOI:** 10.3390/ijms17060918

**Published:** 2016-06-10

**Authors:** Marcio M. Ladeira, Jon P. Schoonmaker, Mateus P. Gionbelli, Júlio C. O. Dias, Tathyane R. S. Gionbelli, José Rodolfo R. Carvalho, Priscilla D. Teixeira

**Affiliations:** 1 Department of Animal Science, Universidade Federal de Lavras, Lavras 37200-000, Brazil; mateus.pg@dzo.ufla.br (M.P.G.); diasjuliovet@yahoo.com.br (J.C.O.D.); tathytt@yahoo.com.br (T.R.S.G.); jose_rodolfo@zootecnista.com.br (J.R.R.C.); priscilla_zoo@yahoo.com.br (P.D.T.); 2 Department of Animal Science, Purdue University, West Lafayette, IN 47906, USA; jschoonm@purdue.edu

**Keywords:** gene expression, transcription factors, lipogenesis, PPAR, *SREBF1*

## Abstract

The objective of the present review is to discuss the results of published studies that show how nutrition affects the expression of genes involved in lipid metabolism and how diet manipulation might change marbling and composition of fat in beef. Several key points in the synthesis of fat in cattle take place at the molecular level, and the association of nutritional factors with the modulation of this metabolism is one of the recent targets of nutrigenomic research. Within this context, special attention has been paid to the study of nuclear receptors associated with fatty acid metabolism. Among the transcription factors involved in lipid metabolism, the peroxisome proliferator-activated receptors (PPARs) and sterol regulatory element-binding proteins (SREBPs) stand out. The mRNA synthesis of these transcription factors is regulated by nutrients, and their metabolic action might be potentiated by diet components and change lipogenesis in muscle. Among the options for dietary manipulation with the objective to modulate lipogenesis, the use of different sources of polyunsaturated fatty acids, starch concentrations, forage ratios and vitamins stand out. Therefore, special care must be exercised in feedlot feed management, mainly when the goal is to produce high marbling beef.

## 1. Introduction

The amount, distribution and composition of fat in beef are some of the factors that exert the greatest impact on the organoleptic and nutritional quality of this food. Procedures to modify and control the formation of adipose tissue in cattle have been largely investigated in animal nutrition and growth in the past decades. Understanding the physiology of the formation and composition of fat in cattle might result in countless possibilities towards guiding nutrition and the production of high-quality beef. Several studies have found correlations between marbling score and meat flavor. Thus, nutrition and management strategies able to increase the intramuscular fat content might contribute to increasing the added value of beef.

The formation and composition of fat in cattle and other ruminants is a complex process with multifactorial regulation. Genetic factors, age at slaughter, growth rate and sex are the parameters that exert the most impact on the amount and composition of animal fat. In addition, nutritional factors act differently on lipid synthesis of ruminants and non-ruminants. In the latter, carcass fat profile is similar to the profile of fatty acids consumed [1], while, in the former, ruminal biohydrogenation and fatty acid tissue metabolism (mRNA synthesis, enzyme activity, *etc.*) interfere considerably with synthesis and the profile of fatty acids that composes the meat fat [2].

Two pathways of action should be studied with regard to lipid metabolism and beef quality. One pathway is related to knowledge on the ruminal biohydrogenation of dietary fatty acids, and, consequently, on the amount and concentration of fatty acids that are absorbed and transported to the tissues. The other pathway is related to the action of fatty acids as metabolic modifiers, either by directly altering the synthesis and deposition of fatty acids in the tissues or by affecting biological processes in the animals, such as the modulation of gene expression in ruminants. In either case, understanding the action of fatty acids in animal metabolism demands adequate knowledge of the molecular processes involved in the synthesis and utilization of fatty acids in the tissues. The studies addressing these subjects usually associate gene expression and nutrition; therefore, they are called nutrigenomics or functional genomics.

Before discussing in detail how some nutrients, such as fatty acids, might change the expression of definite genes, the main processes involved in adipogenesis and lipid metabolism will be briefly reviewed, as several of the investigated genes encode enzymes or are transcription factors that modulate the expression of genes involved in lipid metabolism.

## 2. Adipogenesis

Adipose tissue originates in the embryonic mesoderm and contains a variety of cells, including mesenchymal stem cells (MSC), preadipocytes, fibroblasts, and adipocytes. Adipogenesis is an inclusive term describing the commitment of progenitors (MSC) to pre-adipocytes (determination), proliferation of pre-adipocytes, differentiation of pre-adipocytes into adipocytes, and conversion of cells into lipid-assimilating cells found within fat tissue [3,4]. Adipogenesis is initiated around mid-gestation in ruminant animals [5,6,7], is most active perinatally, and continues throughout the animal’s lifetime [8]. Prenatal adipogenesis occurs in an asynchronous manner [9], whereas postnatal adipogenesis occurs more rapidly depending on availability of energy and regulatory mechanisms [10]. Adipose tissue is a connective tissue [11] derived from multipotent MSC that, like muscle, bone and cartilage are generally believed to have mesodermal origin. Mesenchymal stem cells are abundant during early developmental stages, particularly in the fetus and neonate, but their occurrence diminishes as animals become older.

### 2.1. Commitment

Currently, the mechanisms controlling adipogenesis in fetal and postnatal skeletal muscle *in vivo* remain poorly defined, although numerous *in vitro* cell culture studies suggest that peroxisome proliferator-activated receptor (PPARγ) and CCAAT-enhancer-binding proteins (C/EBP) are crucial factors controlling adipogenesis from commitment of multipotent stem cells to differentiation into adipocytes [12,13,14]. The current thought is that MSC at the initial stage of determination give rise to myogenic factor five expressing (myf5(+)) and non-expressing (myf5(−)) cells [15]. Myf5 is a crucial early myogenic transcription factor, expression of which is highly specific to committed skeletal myoblastic cells [16]. Muscle and brown adipocytes develop from *myf5*(+) cells, whereas chondrocytes, osteoblasts, fibroblasts and white adipoblasts develop from myf5(−) cells [4,17]. The further commitment of myf5(+) and myf5(−) cells to myogenic, adipogenic, or osteogenic cells is controlled by different groups of protein such as the zinc finger protein (ZFP) family, the wingless (Wnt) protein family, the hedgehog protein family, the bone morphogenic protein (BMP) family, and the nuclear hormone superfamily [18,19,20]. The outcomes of different transcription factors depend on relative concentration, stage of differentiation, cell to cell interactions, and the nature of the extracellular matrix. The exact stage at which they act is not well defined in the literature and needs further research to elucidate the precise role of these signals.

Zinc finger proteins are the largest transcription factor family in mammals [21] and contain one or more zinc finger motif(s) that regulate diverse growth and developmental processes, including adipogenesis, through DNA/RNA binding, protein–protein interactions, transcription activation, and regulation of apoptosis [22]. Zinc finger proteins, notably Zfp423, Zfp467, and Zfp521 control adipogenesis by activating/inhibiting/recruiting key modulators of adipogenesis (PPARγ, C/EBPs, Pref-1) or other transcriptional factors [20]. Zfp521 inhibits PPARγ and adipocyte commitment and promotes bone development. Repression of Zfp521 is one of the earliest known events in commitment of stem cells into white adipocytes [23]. Zfp423 promotes commitment of MSC to the adipocyte lineage by activating transcription of *PPARG* [23]. Zfp467 promotes adipocyte commitment and suppresses osteoblast differentiation [20].

**Figure 1:**
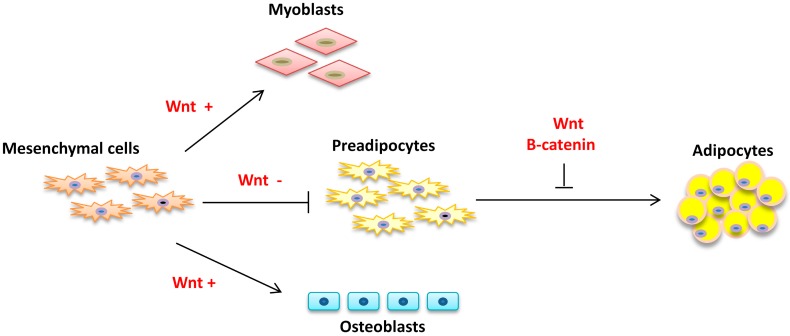
Mesenchymal stem cell fate is regulated by WNT signaling. The activation of WNT/β-catenin signaling order the differentiation of mesenchymal cells into myoblasts and osteoblasts while the commitment of mesenchymal cells to the adopocytic lineage is suppressed.

Wingless/int (Wnt) is a 19-member family of secreted signaling proteins playing a major role in cell fate commitment, embryonic development, and differentiation [24,25]. Wnt proteins suppress adipogenic differentiation and favors myogenic and osteogenic differentiation ([26] Figure 1). In addition, activation of the hedgehog (Hh) pathway blocks formation of fat tissue through suppression of PPARγ promoter activity [27].

BMPs are the members of transforming growth factor β (TGFβ) super family and play critical role in the commitment of MSCs into cell lineages. There are 14 members in the BMP family (BMP-2 to BMP-15). BMP4 stimulates the differentiation of MSC to adipocyte lineage, BMP2 promotes the osteogenic lineage, and BMP7 plays a crucial role in brown adipocyte differentiation [28]. The primary function of many proadipogenic factors (*i.e.*, b-catenin and preadipocyte factor-1) is repression of genes inhibiting adipogenesis. BMP4 dissociates a complex of Wnt proteins that suppress *PPARG* expression and adipogenesis [29] (Figure 1).

Fibroblast growth factors (FGFs) are a family of key extracellular signaling peptides which regulate many biological processes, including cell proliferation and control of embryonic development [30]. FGF10 mRNA is expressed primarily in white adipocytes and may act as a growth factor for white pre-adipocytes [31,32]. Brown adipocyte lineage commitment and differentiation is controlled by PR domain containing protein 16 (PRDM16) and PPARγ. PRDM16 acts as a switch between myogenic lineage and brown adipocytes [33,34]. PRDM16 expressing cells do not undergo myogenic lineage differentiation. PRDM16 expression also induces PPARγ co-activator 1α (*PPARGC1A*) gene expression, which is specific to brown adipocytes. It co-activates the transcriptional activity of Peroxisome Proliferator-Activated Receptor Gamma Co-activator 1 Alpha (PGC1α) and PGC1β as well as PPARα and PPARγ through direct interaction [35].

More recently, preadipocyte factor 1 (*Pref-1*), also known as Delta-like 1 homolog (*Dlk1*), has been shown to play a potential role in early commitment of stem-like cells to the adipocyte lineage [36]. Established as a transmembrane protein that is a member of epidermal growth factor-like protein family, Pref-1 also acts to regulate the cell’s entry into G1/S-phase of the cell cycle and subsequently inhibits proliferation and differentiation [37,38].

### 2.2. Proliferation and Differentiation

The next stage of the process of adipogenesis is the re-entry of growth-arrested preadipocytes into the cell cycle and subsequent proliferation [39]. The committed preadipocytes maintain the capacity to proliferate, but have to withdraw from the cell cycle to undergo differentiation. Cell cycle arrest is a key stage necessary for adipocyte differentiation [40]. At this stage, a transient expression of *CEBPB* (the gene responsible to encode CCAAT/enhancer binding protein β), is rapidly induced, consequently initiating adipocyte differentiation [41]. The subsequent expression of *PPARG* and *CEBPA* transactivates adipocyte specific cell cycle arrest genes and ends proliferation. Sterol regulatory element binding protein-1c (SREBP-1c)/adipocyte determination and differentiation factor-1 (ADD1) is a transcription factor that is involved in cholesterol metabolism and adipocyte specific gene expression [42,43,44,45] that is also induced early during adipocyte differentiation.

The late stage of differentiation is characterized by the increase in expression of proteins involved in *de novo* lipogenesis such as FABP4, adiponectin, leptin, *etc.* [40,41,46]. In addition, the activity of enzymes involved in triacylglycerol metabolism such as glycerol-3-phosphate acyltransferase, glycerol-3-phosphate dehydrogenase, *etc.* increases 10–100-fold [47]. The adipocytes acquire sensitivity to insulin during late stage of differentiation as a result of an increase in insulin receptor numbers and glucose transporters (GLUT4). Also during differentiation, cells convert from fibroblast to spherical morphology. This is accompanied by dramatic changes in the cytoskeleton and extracellular matrix component (ECM). Decreases in actin and tubulin expression as well as fibroblast expressed type-I and III procollagen are also seen in the early stage of differentiation [48,49]. There is also a dramatic decrease in preadipocyte factor-1 (pref1) expression as cells differentiate from preadipocytes to mature adipocytes [37,50].

In adults, it is thought that adipose tissue mesenchymal stem cells serve as a reservoir and allow the continued renewal of precursor cells that can differentiate into adipocytes [51]. Differentiated white (pre)adipocytes secrete BMP4, which starts a cascade of events that leads to PPARγ activation and subsequent adipogenesis [52].

### 2.3. Intramuscular Fat

Despite similar morphological appearance of white fat tissue in every part of the body, there are major regional differences spanning from distinct gene expression profiles to distinct adipokine production [53]. Microarray molecular analyses have confirmed that both human and mouse white fat tissues from different anatomical locations differ in a large number of expressed genes, including developmental patterning genes [54,55,56]. Intramuscular fat is considered to be connective tissue, and its development is inseparable from fibrogenesis [57]. Fibrogenesis occurs throughout an animals lifetime, but is most active in the fetus during late gestation when primordial perimysium and epimysium of muscle bundles are synthesized [8].

Myogenic progenitors develop into satellite cells and muscle fibers, whereas non-myogenic progenitor cells develop into the stromal-vascular fraction within skeletal muscle where adipocytes, fibroblasts and mesenchymal progenitor cells are located [58]. These non-myogenic progenitors, so-called “fibro-adipogenic precursor” (FAP), have adipogenic and fibrogenic capacity, as well as osteogenic and chondrogenic potential [59,60]. These cells are mainly located in the stromal-vascular fraction of skeletal muscle and can be distinguished from myogenic satellite cells by the expression of platelet-derived growth factor receptor α (*PDGFRα*) [59,61,62,63]. When muscle is damaged, FAPs respond to local cytokine production by proliferating, clearing necrotic debris, and supporting myogenesis [59,64]. Thus, FAPs play an important role in normal physiology and may contribute to marbling potential of beef cattle.

## 3. Lipogenesis

Lipogenesis is a physiological process of endogenous fatty acid synthesis that increases inversely to muscle tissue development. Therefore, after puberty and sexual maturity, as muscle growth decreases, adipose tissue increases [65]. According to Pethick *et al.* [66], fat deposition is not homogeneous throughout the animal body; the first observed depot to form is internal fat (abdominal, renal-inguinal and pelvic), followed by intermuscular, subcutaneous and intramuscular fat or marbling.

For fat synthesis to occur, triglycerides must be incorporated into the animal adipose tissue, after the absorption of dietary fatty acids or *de novo* synthesis of other fatty acids [67]. The main factor in the control of fat deposition rate is the animal’s nutritional status, as acetate, a volatile fatty acid produced in ruminal fermentation, is the main precursor for the synthesis of fatty acids among ruminants [68]. Following its absorption across the ruminal epithelium and distribution to the peripheral tissues, acetate is converted in adipose cells into acetyl-CoA through the action of the enzyme acetyl-CoA synthetase [69]. The gene that encodes this enzyme is known as acyl-CoA synthetase short-chain family member 2 (*ACSS2*). This step also occurs with dietary long-chain fatty acids.

**Figure 2:**
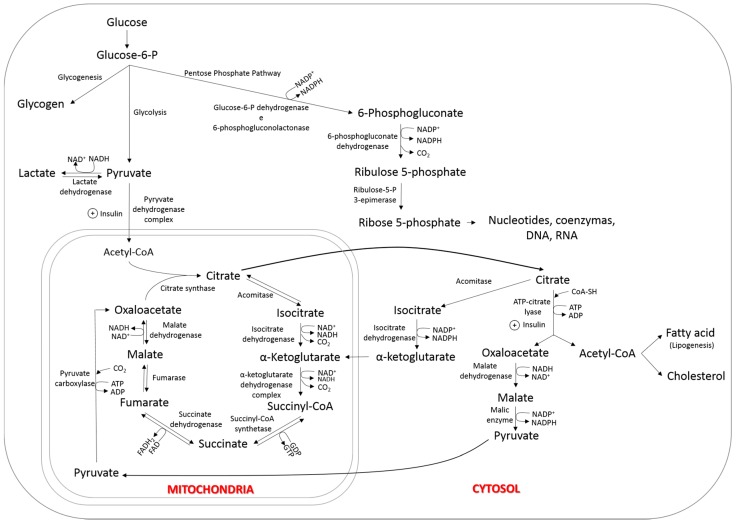
Synthesis of fatty acid from glucose in adipocytes. NAD^+^: nicotinamide adenine dinucleotide oxidized; NADH: nicotinamide adenine dinucleotide reduced; NADP^+^: nicotinamide adenine dinucleotide phosphate oxidized; NADPH: nicotinamide adenine dinucleotide phosphate reduced; CO_2_: carbon dioxide; CoA-SH: coenzyme A; ATP: adenosine triphosphate; ADP: adenosine diphosphate; DNA: deoxyribonucleic acid; RNA: ribonucleic acid; GTP: guanosine triphosphate; GDP: guanosine diphosphate; FAD: flavin adenine dinucleotide oxidized; FADH_2_: flavin adenine dinucleotide reduzed.

The presence of acetyl-CoA and reduced nicotinamide adenine dinucleotide phosphate (NADPH) is essential for *de novo* fatty acid synthesis. In addition to acetate, another source of acetyl-CoA is the pyruvate produced through glycolysis, following decarboxylation in the mitochondria. However, as lipogenesis takes place in the cytosol, acetyl-CoA, which is not permeable through the mitochondrial membrane, is transported as citrate to the extracellular compartment, where it is cleaved by the enzyme citrate lyase into oxaloacetate and acetyl-CoA (Figure 2). NADPH, in turn, is synthesized in the pentose phosphate pathway, and, in the case of ruminants, also through the transformation of citrate into α-ketoglutarate in the cytosol, which then re-enters the mitochondria [70]. In non-ruminant animals, citrate lyase, malate dehydrogenase and malic enzyme are responsible for the production of more NADPH molecules from cytosolic citrate [68].

The next step in the *de novo* synthesis of fatty acids consists of the carboxylation of acetyl-CoA to form malonyl-CoA through the action of the enzyme acetyl-CoA carboxylase, which is encoded by the acetyl-CoA carboxylase alpha (*ACACA*) gene. Next, through the action of the fatty acid synthase multienzyme complex encoded by the fatty acid synthase (*FASN*) gene, another acetyl-CoA molecule is united to malonyl-CoA molecules through multiple serial enzymatic reactions, resulting in the synthesis of long-chain fatty acids. For example, following seven redox reactions, palmitic acid (C16:O) is formed, which is the main fatty acid produced in the animal body [71,72]. According to Ward *et al.* [73] and Underwood *et al.* [74], cattle with higher amounts of marbling have higher activation rates of acetyl-CoA synthetase and the fatty acid synthetase multienzyme complex.

Regarding lipogenesis regulation, the synthesis of the mRNA that encodes these enzymes and their activity are considered essential for *de novo* synthesis of fatty acids [75]. In addition, insulin is one of the main regulatory hormones, as it activates acetyl-CoA carboxylase, citrate lyase and the pyruvate dehydrogenase complex [76,77,78]. Baldwin *et al.* [79], working with abomasal and ruminal infusions of carbohydrates, found that abomasal infusion of glucose increased the transcription of the *FASN* and *ACACA* genes. According to the literature, glucose stimulates the expression of genes that encode lipogenic enzymes, such as *FASN* and *ACACA*, in the adipose tissue of rats when the intracellular glucose-6-phosphate levels are elevated [80].

Palmitic acid is the final product of fatty acid synthesis. However, its chain might undergo elongation, or it can be converted into unsaturated fatty acids through the action of the enzyme stearoyl-CoA desaturase, which might influence the taste, color and nutraceutical properties of the meat [2,81,82]. The gene that encodes stearoyl-CoA desaturase is known as stearoyl-CoA desaturase (*SCD1*), with several studies published in the literature reporting that its expression is significantly influenced by diet [83,84,85,86].

Finally, butyrate is considered a substitute for acetyl-CoA in fatty acid synthesis by the adipose tissue, especially long-chain fatty acids [87]. In addition, propionate and lactate, which are considered gluconeogenic organic acids, might be indirectly used for the synthesis of fatty acids [88], as is discussed next.

## 4. Lipogenesis and Marbling

As mentioned earlier, adipogenesis is initiated around mid-gestation in ruminants and, therefore, nutrition status of the dam may impact fat deposition during finishing phase. According to Schoonmaker [89], marbling results from not only the size but also the number of intramuscular adipocytes, while Du *et al.* [90] observed that nutrition in the fetal and early stages of life exerts a considerable impact on adipocyte hyperplasia.

In general, the size of intramuscular fat depot in adult cattle results from the balance between the synthesis and degradation of triglycerides [91]. In addition, the rate of deposition of intramuscular fat depends not only on the number and intrinsic activity of the intramuscular adipocytes but also on the muscle growth rate and the metabolic activities of other organs [92].

For the deposition of intramuscular fat to occur in finishing cattle, the net energy consumed must surpass requirements; thus, the degree of marbling varies as a function of the energy content in the diet [93]. While high plasma levels of acetate promote greater fatty acid formation in extramuscular adipocytes, propionate and lactate might be precursors for the synthesis of intramuscular fatty acids in ruminants, as they are converted into acetyl-CoA and enter the TCA cycle (Figure 2). Propionate is converted into glucose in the liver, which might subsequently enter the glycolytic pathway, enter the TCA cycle, and become a carbon donor in *de novo* fatty acid synthesis. According to Gilbert *et al.* [94], intramuscular adipose tissue uses a high proportion of glucose for fatty acid synthesis, while the subcutaneous adipose tissue primarily utilizes acetate for lipid synthesis. These authors further reported that intramuscular adipose tissue, compared to subcutaneous fat, is more sensitive to insulin. Thus, foodstuffs that increase propionate production, such as maize and other grains, have higher glycogenic and insulinogenic capacity, which might increase the deposition of intramuscular fat. Chung *et al.* [95] also observed that glucose plays a relevant role as carbon donor in the *de novo* synthesis of fatty acids in the intramuscular fat of cattle.

Therefore, the manipulation of diets to increase the glucose and insulin supply in ruminants represents an interesting strategy to increase the deposition of intramuscular fat. However, according to Rhoades *et al.* [96], the source of dietary energy used might alter insulin sensitivity, causing the tissues to become resistant to the action of this hormone and thus inhibit lipogenesis. This mechanism of resistance was described by Tardif *et al.* [97], who demonstrated that ketone accumulation interrupted insulin signal transduction and reduced the migration of glucose transporters to the cell surface. Such a decrease in glucose transporters would reduce insulin-stimulated glucose uptake, thus limiting the rate of glucose utilization.

Therefore, the use of diets with high concentrate content, resulting in the generation of higher amounts of propionate in addition to higher insulin and circulating glucose concentrations, might increase the deposition of intramuscular fat in beef [98,99,100].

### Nutrigenomics and Circulating Glucose

Several studies sought to establish whether nutrition might alter the expression of genes that encode enzymes involved in the digestion of starch and glucose transporters, thereby increasing circulating glucose. Swanson *et al.* [101] found higher levels and activity of pancreatic α-amylase in lambs fed high-starch diets; however, expression of the alpha 2B amylase (*AMY2B*) gene tended to be lower in lambs fed high-starch compared to animals fed a low-starch diet. These authors observed that the mechanisms of regulation of pancreatic α-amylase in ruminants are very complex and likely regulated by both transcriptional and post-transcriptional events.

In a study conducted with steers, Swanson *et al.* [102] assessed the effects of abomasal infusion of partially hydrolyzed starch and/or casein. The results indicated a tendency for a reduction in *AMY2B* expression in conjunction with decreased synthesis and activity of α-amylase in the animals given partially hydrolyzed starch. Therefore suggesting that there is an inverse relationship between intestinal starch flow and *AMY2B* expression.

Following post-ruminal starch digestion and glucose release into the intestinal lumen, glucose must be absorbed by the enterocytes. According to Kellett *et al.* [103], there are at least three membrane monosaccharide transport proteins in mammals. Ferraris and Diamond [104] showed that nutrients regulate intestinal monosaccharide absorption in many species and that the activity and expression of the genes that encode the glucose transporters might be modulated based on the diets that are used.

The main monosaccharide transporter is the sodium-glucose linked transporter 1 (SGLT1) and is encoded by the solute carrier family 5 (sodium/glucose cotransporter), member 1 (*SLC5A1*) gene. This sodium-dependent glucose transporter is able to transport glucose and most monosaccharides, except for fructose, across the enterocyte brush border. The solute carrier family 2 (facilitated glucose/fructose transporter), member 5 (GLUT5) transports fructose only across the brush border, while the solute carrier family 2 (facilitated glucose transporter), member 2 (GLUT2) transports glucose, fructose and all other monosaccharides across the enterocyte brush border and basolateral membrane [105].

In a study conducted to assess abomasal and ruminal infusions of hydrolyzed starch on monosaccharide transporter mRNA synthesis in the intestine, Liao *et al.* [105] found that ruminal infusion of hydrolyzed starch increased duodenal expression of *SLC5A1* by approximately 64%. In turn, Guimarães *et al.* [106] did not find any effect of post-ruminal starch and casein infusions on *SLC5A1* abundance in the small intestines of Holstein steers. However, the results showed greater gene expression in the middle and end of the jejunum, independent of the treatment. A similar finding was reported by Rodriguez *et al.* [107], who worked with crossbred steers given an abomasal starch and glucose infusion and a ruminal starch infusion.

## 5. Transcription Factors and Lipid Metabolism in Beef Cattle

Several key events involved in the synthesis of fat in the animal tissues take place at the molecular level. The association of nutritional factors with the modulation of fat metabolism is one of the recent targets of nutrigenomic studies conducted with beef cattle. Within this context, special attention has been given to the study of nuclear receptors related to fatty acid metabolism [108,109,110,111,112,113]. Those receptors form an intracellular protein receptor superfamily included within the transcription factors class. Upon binding to the DNA, those factors allow RNA polymerase binding and transcription initiation. Such factors participate in several physiological functions, including homeostasis, reproduction, growth, differentiation, morphogenesis, apoptosis and metabolism [114].

**Figure 3:**
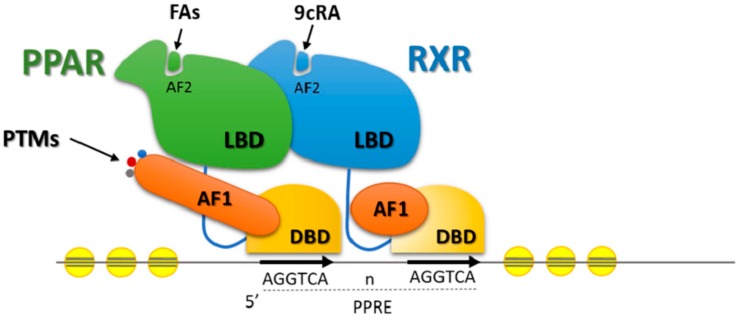
Diagram representing the binding of a nuclear receptor to the DNA. PPAR: peroxisome proliferator-activated receptor, RXR: retinoid X receptor, LBD: ligand-binding domain, PTMs: post-translational modifications, 9cRA: 9-cis retinoic acid, FAs: fatty acids, AF: activation function, DBD: DNA-binding domain, PPRE: peroxisome proliferator response elements.

Among the transcription factors involved in lipid metabolism PPARs and SREBPs stand out. PPARs are a family of nuclear receptors that bind to fatty acids and perform significant functions in the regulation of nutrient metabolism and energy homeostasis [115]. PPAR isoforms work as heterodimers with retinoid X receptor (RXR), and together, both bind to a specific DNA sequence in the promoter region of the target gene, thus inducing or repressing its expression (Figure 3; [116]).

There are three PPAR isoforms, α, γ and β, which differ in terms of target tissue, physiological properties and developmental stage of tissues [117,118]. PPARγ is highly expressed in adipocytes and less in the muscle [119] and plays a crucial role in the control of adipogenesis, lipogenesis and insulin sensitivity [120]. PPARα is highly expressed in the liver, followed by the small intestine, adipose tissue and heart [121], while PPARβ is distributed throughout the body. In the liver, PPARα plays a key role in fatty acid oxidation [122] by inducing the expression of long-chain fatty acid transporter proteins and other enzymes involved in peroxisomal β-oxidation.

PPARγ is the main regulator of fatty acid storage and adipogenesis, as it binds to the genes associated with lipid metabolism, including those that encode fatty acid-binding protein (FABP), acyl-CoA synthetase long-chain family member 1 (*ACSL1*) and lipoprotein lipase (LPL) [123]. Based on this behavior, PPARγ plays a key role in ruminant research, especially because it is associated with candidate genes for marbling regulation [124]. Like the other nuclear receptors, PPARγ also binds to and becomes activated by lipophilic molecules (fatty acids) causing regulation of transcription. PPARγ is able to bind to two different fatty acids at once and is not specific for a single fatty acid, denoting its ability to bind a mixture of fatty acid molecules [125]. In addition to lipogenesis, PPARγ might play an essential role in long-chain fatty acid oxidation by controlling the expression of carnitine palmitoyltransferase 2 (CPT2) and carnitine acetyltransferase (CRAT), genes that are involved in the entry of long-chain fatty acids into the mitochondria [126].

SREBP has a crucial role in energy homeostasis, promoting glycolysis, lipogenesis and adipogenesis [127]. The SREBP family has three members: 1a, 1c and 2. SREBP-1c is encoded by the sterol regulatory element-binding transcription factor 1 (*SREBF1*) gene and seems to act more specifically on the genes involved in fatty acid synthesis [128], while SREBP-2 has greater influence on the regulation of the expression of cholesterogenic genes. SREBP-1c was identified in white adipose tissue and was initially named adipocyte determination and differentiation factor (ADD-1) [129]; it is also expressed in the liver.

Insulin is the main metabolic signal that stimulates and regulates *SREBF1* expression, while glucagon represses it [130]. Most of the lipogenic effects of insulin depend on *SREBF1* expression and the subsequent stimulation of the fatty acid synthesis pathway [130]. *SREBF1* expression is also stimulated by the liver X receptor (LXR), which controls and protects cells against cholesterol overload.

**Figure 4:**
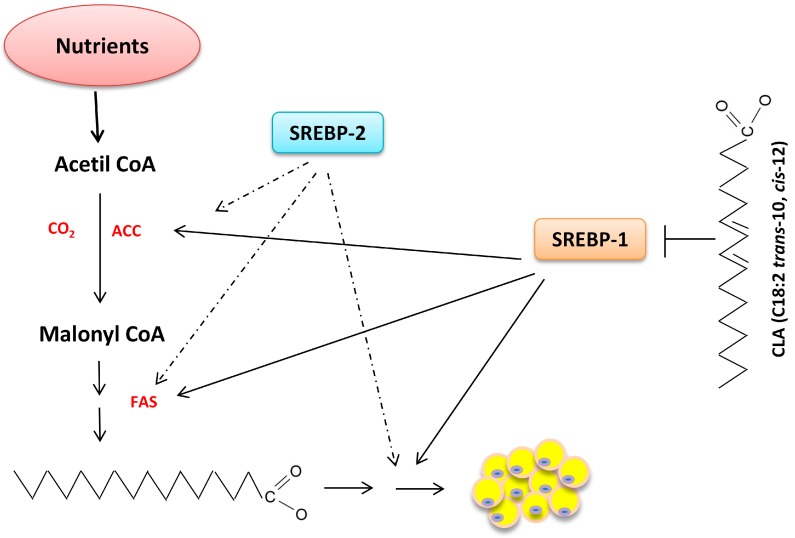
Proposed mechanism for the effect of fatty acids on the regulation of key enzymes in lipogenesis via SREBPs (solid arrows indicate greater effects; dotted arrows indicate lesser effects). SREBP: sterol regulatory element-binding protein, ACC: acetyl-CoA carboxylase, FAS: fatty acid synthase, CLA: conjugated linoleic acid.

The mechanisms of action of SREBPs in the activation and suppression of lipogenesis pathways are depicted in Figure 4. According to previous reports, high levels of polyunsaturated fatty acids (PUFAs), such as *trans*-10 fatty acids, are involved in the reduction of SREBP-1c concentration [131]. In addition, according to Botolin *et al.* [132], *n*-3 PUFA suppresses SREBP-1 nuclear content in the rat liver through 26S proteasome- and Erk-dependent pathways. In mice, CLA also reduced adipose tissue by apoptosis and results in lipodystrophy [133].

### Effects of Nutrients on Transcription Factors Gene Expression

The expression, and consequently the action, of transcription factors depend on the animals’ physiological conditions and developmental stage, whereby the corresponding genes are expressed only at the appropriate times. In addition, definite nutrients, such as long-chain fatty acids, are able to regulate the expression of transcription factors. According to Bionaz *et al.* [111], several fatty acids might, to varying degrees, activate various PPAR isotypes, with PUFAs showing more affinity for PPARα, which is encoded by the peroxisome proliferator-activated receptor alpha (*PPARA*) gene [134].

Thus, Oliveira *et al.* [85] reported greater *PPARA* expression in animals fed ground soybean and monensin, which was associated with an increase in ruminal unsaturated fatty acid biohydrogenation, as the use of ground oilseeds increases fatty acid availability. Teixeira [135] found that *PPARA* expression was higher in animals fed whole shelled corn diet without forage compared to ground corn with forage, which was due to the higher PUFA concentration in that type of feed. In addition, the whole shelled corn diet without forage was associated with an increased concentration of the conjugated linoleic acid (CLA) C18:2 *trans*-10, *cis*-12 compared to ground corn with forage. In this study, 28 bulls were used in a completely randomized design and arranged as a 2 × 2 factorial (two breeds: Angus and Nellore; and two diets: whole shelled corn diet and ground corn diet). The ground diet had 30% of corn silage and 70% of a concentrate based on corn and soybean meal. The whole shelled corn diet had 85% whole shelled corn and 15% of a pellet based on soybean meal and minerals. The feedlot lasted 81 days and bulls were slaughtered with 500 kg. Bionaz *et al.* [111], reported that C18:2 *trans*-10, *cis*-12 had an agonistic effect on PPARα. In addition, Brown *et al.* [136] found that, in humans, C18:2 *trans*-10, *cis*-12 decreases the expression of peroxisome proliferator-activated receptor γ (*PPARG*) and consequently reduces glucose and lipid absorption and oxidation and pre-adipocyte differentiation. Therefore, this CLA might have an antagonistic effect on the expression of PPAR isoforms.

Sanosaka *et al.* [137] showed that increased oleic acid concentration in the culture medium was associated with increased *PPARG* expression in porcine pre-adipocytes. According to Smith *et al.* [138], the differentiation of bovine pre-adipocytes might be strongly stimulated through the addition of PPAR agonists such as insulin and dexamethasone.

With regard to the transcription factor SREBP-1c, reports in the literature indicate that grain-rich diets decrease the expression of *SREBF1* in the mammary gland due to a decrease in ruminal pH, which might alter the biohydrogenation pathways and increase C18:2 *trans*-10, *cis*-12 synthesis [139]. The increase in this intermediary decreases *SREBF1* mRNA levels, consequently decreasing the activity of the enzymes involved in *de novo* synthesis [131].

**Figure 5:**
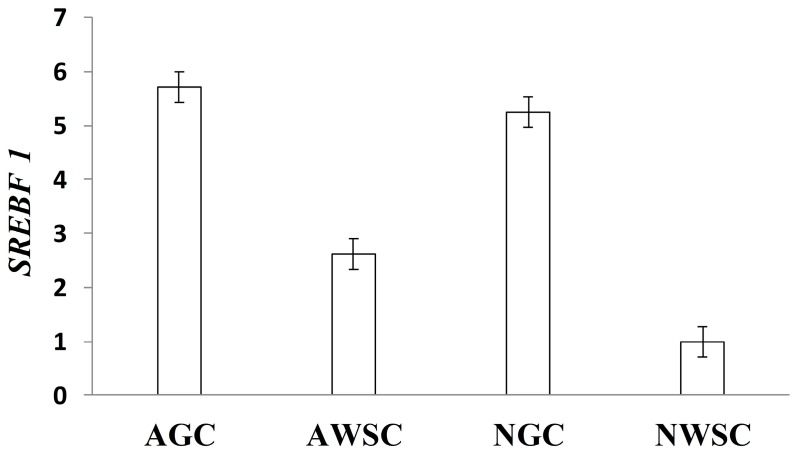
Relative expression of *SREBF1* in the *Longissimus dorsi* muscle of Angus (A) and Nellore (N) bulls fed a ground corn diet (GC: 30% corn silage plus 70% concentrate based on ground corn and soybean meal) and whole shelled corn (WSC: 85% whole shelled corn plus 15% proteic-mineral supplement). Source: Teixeira [135].

Additionally, Teixeira [135] (Figure 5) detected decreased *SREBF1* expression in the muscles of cattle fed a whole shelled corn diet without forage. Such a diet induced a more pronounced fall in ruminal pH, which favored the deposition of C18:2 *trans*-10, *cis*-12. In a study conducted with pigs, Brandebourg and Hu [140] also found that C18:2 *trans*-10, *cis*-12 reduced *SREBF1* expression in pre-adipocytes.

## 6. Nutrigenomics and Lipid Metabolism

### 6.1. Tissue Uptake of Fatty Acids

Triglycerides stored in adipocytes might originate either from the uptake of fatty acids from the blood or from *de novo* synthesis. For uptake to occur, the circulating triglycerides present in lipoproteins, such as chylomicrons, must first undergo the action of the enzyme LPL and then be carried by fatty acid-binding protein 4 (FABP4), which is responsible for the transport of fatty acids into cells [141].

Confirming the interaction between *LPL* and *FABP4* genes, Teixeira [135] found positive correlations between them and greater expression of both in the muscle of Nellore cattle fed maize silage plus concentrate. Costa *et al.* [112] found that animals fed a diet with a high forage percentage tended to exhibit higher *LPL* expression in the muscle. In contrast, Zhang *et al.* [86] found greater *LPL* and *FABP* expression in high-energy diets compared to low-energy diets. According to these authors, increased dietary energy might improve nutrient digestion and absorption, which would stimulate the expression of the genes responsible for fatty acid transport. Similarly, Graugnard *et al.* [83] found higher *FABP* expression in animals fed a high-starch diet, and Peng *et al.* [142] found higher *LPL* expression in animals fed a diet with higher energy density.

The findings reported by Joseph *et al.* [143] suggest that corn oil supplementation regulates *LPL* expression by increasing the amount of fatty acids available for absorption. Waylan *et al.* [144] found that flaxseed supplementation (5% of the dietary dry mass) was associated with increased *LPL* expression in the muscle tissue of finishing cattle compared to the control diet.

Oliveira *et al.* [85] reported greater *LPL* and *FABP4* gene expression in animals fed soybean compared to animals fed rumen-protected fat and explained that this difference was due to the difference in the fatty acid compositions of the investigated feeds and changes in the expression of the transcription factor *PPARA*. This mode of action is supported by the results of a comparative analysis of *FABP4* promoter regions among mammals demonstrating the presence of two PPARα-binding sites in human, rat, pig, dog and bovine cells [145,146].

Therefore, studies have shown that *LPL* and *FABP4* expression depends on the energy level of the diet and that changes in the composition of the dietary fatty acids that are absorbed in the small intestine might alter the expression of *PPARA* as a function of the positive correlation between those genes. Therefore, when the expression of one gene increases, the others exhibit a similar behavior.

### 6.2. Synthesis of Fatty Acids

As previously mentioned, acetyl-CoA carboxylase (ACC) and the fatty acid synthase complex (FAS) are the enzymes involved in *de novo* synthesis [75]. Following their synthesis or uptake by adipocytes, the fatty acids might be exposed to the action of the enzyme stearoyl-CoA desaturase (SCD).

In mammals, ACC activity is highly regulated by the diet, hormones and other physiological factors [147]. Food intake, especially of low-fat foods, induces ACC synthesis, with a consequent increase in its activity [148]. In fish, ACC was mainly studied due to its role in the biosynthesis of fatty acids in the adipose tissue; such studies were conducted in the liver and in primary hepatocyte cultures [149].

The possible involvement of ACC activity in the formation of marbling in cattle was investigated by Underwood *et al.* [74]. The authors found that ACC inactivation rate was lower in animals with higher marbling amounts. Therefore, post-transcriptional factors play essential roles in ACC regulation. In addition, reports in the literature indicate that *ACACA* gene expression is also influenced by diet. Ladeira [150] found that *ACACA* expression was higher in the muscle of animals fed soybean compared to those fed cottonseed, which might be explained due to the changes in *PPARA* expression and the strong correlation in the mRNA synthesis of both genes.

Zhang *et al.* [86] observed greater *ACACA*, *FASN* and *SCD1* expression in animals fed a high-energy diet. Enzymatic activity also increased in animals fed a high-energy diet. Similarly, Graugnard *et al.* [83] reported greater expression of *ACACA*, *FASN* and *SCD1* genes in animals fed a high-starch diet. According to Ward *et al.* [73], greater *FASN* expression was associated with higher marbling levels in the muscle.

**Figure 6:**
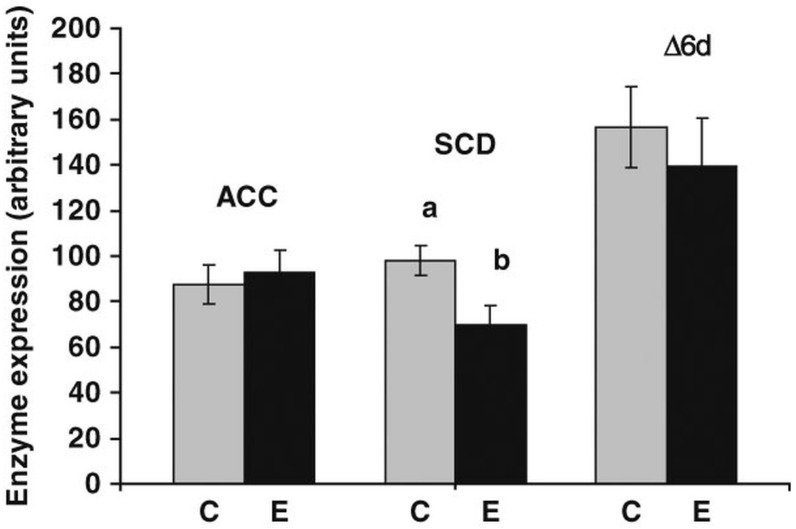
Effects of colza supplementation on the expression of acetyl-CoA carboxylase (*ACACA*), stearoyl-CoA desaturase (*SCD1*) and ∆6-dessaturase (*∆6d*) genes in the *longissimus* muscle. C = control group (maize silage with soybean-based concentrate); E = experimental group (grass silage with linseed oil and rapeseed cake supplemented concentrate). The a and b superscripts means significant difference (*p* < 0.05) between control and experiment group. Source: Herdmann *et al.* [109].

The fact that high-energy diets increase the expression of the genes involved in *de novo* synthesis is reasonable. However, when the increase in the dietary energy is due to higher concentrations of fat, one should be concerned with the fatty acid profile of the source. Waters *et al.* [84] showed that PUFA supplementation inhibited the expression of the gene that encodes SCD in the beef cattle. The authors also observed that the degree of transcription inhibition was associated with PUFA dietary levels. *SCD1* expression was also significantly reduced in the subcutaneous adipose tissue of cattle fed diets with high ω-3 PUFA content, which reduced CLA (C18:2 *cis*-9, *trans*-11) and oleic acid (C18:1 *cis*-9) contents [109] (Figure 6). These findings are consistent with the data in the literature on the inhibitory effects of PUFAs on *SCD1* in other species [151]. Different expression levels of this gene in response to dietary manipulation suggest the existence of a tissue-specific mechanisms and possibly different actions of the transcription factors related to its regulation in ruminants.

Ladeira [150] found greater *SCD1* expression in the muscle of animals fed a cottonseed-based diet compared to the muscle of animals fed ground soybean. In contrast, Yang *et al.* [152] reported that cottonseed oil reduces the activity of SCD due to the presence of sterculic acid in this oilseed. Therefore, the authors suggest that the mechanism of action of sterculic acid present in cottonseed takes place in some post-transcriptional mechanism. Corroborating this assertion, Kadgowda *et al.* [153] showed that sterculic acid directly inhibits enzymatic activity without interfering with *SCD1* gene expression.

Protein supplementation might also influence the expression of the genes involved in *de novo* synthesis (of fat). According to Zhang *et al.* [154], protein supplementation increases *SREBF1*, *ACACA*, *FASN* and *SCD1* expression levels, with greater expression in animals supplemented with 19% crude protein.

Some studies have suggested that *SCD1* expression is regulated by factors associated with SREBP-1c [155,156]. According to Sampath *et al.* [156], changes in *SREBF1* expression might alter SCD synthesis and cause differences in the fatty acid composition of animal adipose tissue. In agreement with the aforementioned studies, Ladeira [150] observed a positive correlation in *SREBF1* and *SCD1* expression. In addition, those genes exhibited greater expression in the muscles of animals fed cottonseed, which might be associated with a higher concentration of ω-6 fatty acids in the muscle, as ω-6 PUFAs are weaker inhibitors of *SREBF1* compared to ω-3 PUFAs [157].

## 7. Final Considerations

To date, the results in the literature indicate that the expression of genes involved in lipid metabolism is influenced by animal nutrition and that diet manipulation might change muscle marbling and molecular composition of fat in beef. Among the options for dietary manipulation, PUFA sources, starch concentration, forage proportions and vitamins stand out. Therefore, special care must be exercised in feedlot feed management, mainly when the goal is to produce high marbling beef.

## References

[1] Nuernberg K., Fischer K., Nuernberg G., Kuechenmeister U., Klosowska D., Eliminowska-Wenda G., Fiedler I., Ender K. (2005). Effects of dietary olive and linseed oil on lipid composition, meat quality, sensory characteristics and muscle structure in pigs. Meat Sci..

[2] Ladeira M.M., Santarosa L.C., Chizzotti M.L., Ramos E.M., Machado Neto O.R., Oliveira D.M., Carvalho J.R., Lopes L.S., Ribeiro J.S. (2014). Fatty acid profile, color and lipid oxidation of meat from young bulls fed ground soybean or rumen protected fat with or without monensin. Meat Sci..

[3] Hausman G.J., Dodson M.V., Ajuwon K., Azain M., Barnes K.M., Guan L.L., Jiang Z., Poulos S.P., Sainz R.D., Smith S. (2009). Board-invited review: The biology and regulation of preadipocytes and adipocytes in meat animals. J. Anim. Sci..

[4] Tseng Y.H., Cypess A.M., Kahn C.R. (2010). Cellular bioenergetics as a target for obesity therapy. Nat. Rev. Drug Discov..

[5] Feve B. (2005). Adipogenesis: Cellular and molecular aspects. Best Pract. Res. Clin. Endocrinol. Metab..

[6] Gnanalingham M.G., Mostyn A., Symonds M.E., Stephenson T. (2005). Ontogeny and nutritional programming of adiposity in sheep: Potential role of glucocorticoid action and uncoupling protein-2. Am. J. Physiol. Regul. Integr. Comp. Physiol..

[7] Muhlhausler B.S., Duffield J.A., McMillen I.C. (2007). Increased maternal nutrition stimulates peroxisome proliferator activated receptor-γ, adiponectin, and leptin messenger ribonucleic acid expression in adipose tissue before birth. Endocrinology.

[8] Du M., Yin J.D., Zhu M.J. (2010). Cellular signaling pathways regulating the initial stage of adipogenesis and marbling of skeletal muscle. Meat Sci..

[9] Hausman G.J., Richardson R.L. (2004). Adipose tissue angiogenesis. J. Anim. Sci..

[10] Azain M.J. (2004). Role of fatty acids in adipocyte growth and development. J. Anim. Sci..

[11] Scanes C.G., Scanes C.G. (2003). Adipose growth. Biology of Growth of Domestic Animals.

[12] Cho Y.C., Jefcoate C.R. (2004). PPARγ1 synthesis and adipogenesis in C3H10T1/2 cells depends on S-phase progression, but does not require mitotic clonal expansion. J. Cell. Biochem..

[13] Giri S., Rattan R., Haq E., Khan M., Yasmin R., Won J.S., Key L., Singh A.K., Singh I. (2006). AICAR inhibits adipocyte differentiation in 3T3L1 and restores metabolic alterations in diet-induced obesity mice model. Nutr. Metab..

[14] Fernyhough M.E., Okine E., Hausman G., Vierck J.L., Dodson M.V. (2007). PPAR gamma and glut-4 expression as developmental regulators/markers for preadipocyte differentiation into an adipocyte. Domest. Anim. Endocrinol..

[15] Kuhn E., Binart N., Lombes M. (2012). Brown, white, beige: The color of fat and new therapeutic perspectives for obesity. Ann. Endocrinol..

[16] Pownall M.E., Gustafsson M.K., Emerson C.P. (2002). Myogenic regulatory factors and the specification of muscle progenitors in vertebrate embryos. Annu. Rev. Cell Dev. Biol..

[17] Fruhbeck G., Becerril S., Sainz N., Garrastachu P., Garcia-Velloso M.J. (2009). Bat: A new target for human obesity?. Trends Pharmacol. Sci..

[18] Busser B.W., Gisselbrecht S.S., Shokri L., Tansey T.R., Gamble C.E., Bulyk M.L., Michelson A.M. (2013). Contribution of distinct homeodomain DNA binding specificities to drosophila embryonic mesodermal cell-specific gene expression programs. PLoS ONE.

[19] Gattu A.K., Swenson E.S., Iwakiri Y., Samuel V.T., Troiano N., Berry R., Church C.D., Rodeheffer M.S., Carpenter T.O., Chung C.H. (2013). Determination of mesenchymal stem cell fate by pigment epithelium-derived factor (PEDF) results in increased adiposity and reduced bone mineral content. Faseb J..

[20] Wei S., Zhang L., Zhou X., Du M., Jiang Z., Hausman G.J., Bergen W.G., Zan L., Dodson M.V. (2013). Emerging roles of zinc finger proteins in regulating adipogenesis. Cell. Mol. Life Sci..

[21] Ganss B., Jheon A. (2004). Zinc finger transcription factors in skeletal development. Crit. Rev. Oral Biol. Med..

[22] Leon O., Roth M. (2000). Zinc fingers: DNA binding and protein-protein interactions. Biol. Res..

[23] Gao H., Mejhert N., Fretz J.A., Arner E., Lorente-Cebrian S., Ehrlund A., Dahlman-Wright K., Gong X.W., Stromblad S., Douagi I. (2014). Early B cell factor 1 regulates adipocyte morphology and lipolysis in white adipose tissue. Cell Metab..

[24] Liu Y., Lv W.T., Yu B.Y., Ju T.T., Yang F.Y., Jiang M.H., Liu Z.H., Xu L., Sun W.J., Huang J.X. (2013). S-adenosylmethionine-induced adipogenesis is accompanied by suppression of WNT/β-catenin and hedgehog signaling pathways. Mol. Cell. Biochem..

[25] Nakamura Y., Hinoi E., Iezaki T., Takada S., Hashizume S., Takahata Y., Tsuruta E., Takahashi S., Yoneda Y. (2013). Repression of adipogenesis through promotion of Wnt/β-catenin signaling by tis7 up-regulated in adipocytes under hypoxia. BBA Mol. Basis Dis..

[26] Christodoulides C., Lagathu C., Sethi J.K., Vidal-Puig A. (2009). Adipogenesis and WNT signalling. Trends Endocrinol. Metab..

[27] Tong Q., Tsai J., Tan G., Dalgin G., Hotamisligil G.S. (2005). Interaction between gata and the C/EBP family of transcription factors is critical in gata-mediated suppression of adipocyte differentiation. Mol. Cell. Biol..

[28] Yoshida H., Kanamori Y., Asano H., Hashimoto O., Murakami M., Kawada T., Matsui T., Funaba M. (2013). Regulation of brown adipogenesis by the TGF-β family: Involvement of Srebp1c in TGF-β- and activin-induced inhibition of adipogenesis. Biochim. Biophys. Acta.

[29] Gupta R.K., Arany Z., Seale P., Mepani R.J., Ye L., Conroe H.M., Roby Y.A., Kulaga H., Reed R.R., Spiegelman B.M. (2010). Transcriptional control of preadipocyte determination by Zfp423. Nature.

[30] Oulion S., Bertrand S., Escriva H. (2012). Evolution of the *FGF* gene family. Int. J. Evol. Biol..

[31] Hotta Y., Nakamura H., Konishi M., Murata Y., Takagi H., Matsumura S., Inoue K., Fushiki T., Itoh N. (2009). Fibroblast growth factor 21 regulates lipolysis in white adipose tissue but is not required for ketogenesis and triglyceride clearance in liver. Endocrinology.

[32] Yamasaki M., Emoto H., Konishi M., Mikami T., Ohuchi H., Nakao K., Itoh N. (1999). FGF-10 is a growth factor for preadipocytes in white adipose tissue. Biochem. Biophs. Res. Commun..

[33] Fruhbeck G., Sesma P., Burrell M.A. (2009). Prdm16: The interconvertible adipo-myocyte switch. Trends Cell Biol..

[34] Seale P., Kajimura S., Yang W., Chin S., Rohas L.M., Uldry M., Tavernier G., Langin D., Spiegelman B.M. (2007). Transcriptional control of brown fat determination by PRDM16. Cell Metab..

[35] Becerril S., Gomez-Ambrosi J., Martin M., Moncada R., Sesma P., Burrell M.A., Fruhbeck G. (2013). Role of PRDM16 in the activation of brown fat programming. Relevance to the development of obesity. Histol. Histopathol..

[36] Hudak C.S., Gulyaeva O., Wang Y.H., Park S.M., Lee L., Kang C., Sul H.S. (2014). Pref-1 marks very early mesenchymal precursors required for adipose tissue development and expansion. Cell Rep..

[37] Hudak C.S., Sul H.S. (2013). Pref-1, a gatekeeper of adipogenesis. Front. Endocrinol..

[38] Traustadottir G.A., Kosmina R., Sheikh S.P., Jensen C.H., Andersen D.C. (2013). Preadipocytes proliferate and differentiate under the guidance of Delta-like 1 homolog (DLK1). Adipocyte.

[39] Camp H.S., Ren D.L., Leff T. (2002). Adipogenesis and fat-cell function in obesity and diabetes. Trends Mol. Med..

[40] Gregoire F.M., Smas C.M., Sul H.S. (1998). Understanding adipocyte differentiation. Physiol. Rev..

[41] Zamani N., Brown C.W. (2011). Emerging roles for the transforming growth factor-β superfamily in regulating adiposity and energy expenditure. Endocr. Rev..

[42] Hao Q., Hansen J.B., Petersen R.K., Hallenborg P., Jorgensen C., Cinti S., Larsen P.J., Steffensen K.R., Wang H., Collins S. (2010). ADD1/SREBP1C activates the PGC1-α promoter in brown adipocytes. Biochim. Biophys. Acta.

[43] Kim J.B., Spiegelman B.M. (1996). ADD1/SREBP1 promotes adipocyte differentiation and gene expression linked to fatty acid metabolism. Genes Dev..

[44] Kim K.H., Song M.J., Yoo E.J., Choe S.S., Park S.D., Kim J.B. (2004). Regulatory role of glycogen synthase kinase 3 for transcriptional activity of ADD1/SREBP1C. J. Biol. Chem..

[45] Seo J.B., Moon H.M., Noh M.J., Lee Y.S., Jeong H.W., Yoo E.J., Kim W.S., Park J., Youn B.S., Kim J.W. (2004). Adipocyte determination- and differentiation-dependent factor 1/sterol regulatory element-binding protein 1c regulates mouse adiponectin expression. J. Biol. Chem..

[46] Cristancho A.G., Lazar M.A. (2011). Forming functional fat: A growing understanding of adipocyte differentiation. Nat. Rev. Mol. Cell Biol..

[47] Chapman A.B., Knight D.M., Dieckmann B.S., Ringold G.M. (1984). Analysis of gene-expression during differentiation of adipogenic cells in culture and hormonal-control of the developmental program. J. Biol. Chem..

[48] Cryer A., van R.L.R. (1982). Characterization of the collagen types synthesized by human and rat adipocyte precursors invitro. Eur. J. Clin. Investig..

[49] Kubo Y., Kaidzu S., Nakajima I., Takenouchi K., Nakamura F. (2000). Organization of extracellular matrix components during differentiation of adipocytes in long-term culture. In Vitro Cell. Dev. Biol. Anim..

[50] Wang Y.H., Kim K.A., Kim J.H., Sul H.S. (2006). Pref-1, a preadipocyte secreted factor that inhibits adipogenesis. J. Nutr..

[51] Bowers R.R., Kim J.W., Otto T.C., Lane M.D. (2006). Stable stem cell commitment to the adipocyte lineage by inhibition of DNA methylation: Role of the *BMP-4* gene. Proc. Natl. Acad. Sci. USA.

[52] Gustafson B., Hedjazifar S., Gogg S., Hammarstedt A., Smith U. (2015). Insulin resistance and impaired adipogenesis. Trends Endocrinol. Metab..

[53] Mueller E. (2014). Understanding the variegation of fat: Novel regulators of adipocyte differentiation and fat tissue biology. BBA Mol. Basis Dis..

[54] Gesta S., Bluher M., Yamamoto Y., Norris A.W., Berndt J., Kralisch S., Boucher J., Lewis C., Kahn C.R. (2006). Evidence for a role of developmental genes in the origin of obesity and body fat distribution. Proc. Natl. Acad. Sci. USA.

[55] Gesta S., Tseng Y.H., Kahn C.R. (2007). Developmental origin of fat: Tracking obesity to its source. Cell.

[56] Karastergiou K., Fried S.K., Xie H., Lee M.J., Divoux A., Rosencrantz M.A., Chang R.J., Smith S.R. (2013). Distinct developmental signatures of human abdominal and gluteal subcutaneous adipose tissue depots. J. Clin. Endocr. Metab..

[57] Miao Z.G., Zhang L.P., Fu X., Yang Q.Y., Zhu M.J., Dodson M.V., Du M. (2016). Invited review: Mesenchymal progenitor cells in intramuscular connective tissue development. Animal.

[58] Du M., Huang Y., Das A.K., Yang Q., Duarte M.S., Dodson M.V., Zhu M.J. (2013). Meat science and muscle biology symposium: Manipulating mesenchymal progenitor cell differentiation to optimize performance and carcass value of beef cattle. J. Anim. Sci..

[59] Joe A.W.B., Yi L., Natarajan A., Le Grand F., So L., Wang J., Rudnicki M.A., Rossi F.M.V. (2010). Muscle injury activates resident fibro/adipogenic progenitors that facilitate myogenesis. Nat. Cell Biol..

[60] Wosczyna M.N., Biswas A.A., Cogswell C.A., Goldhamer D.J. (2012). Multipotent progenitors resident in the skeletal muscle interstitium exhibit robust BMP-dependent osteogenic activity and mediate heterotopic ossification. J. Bone Miner. Res..

[61] Uezumi A., Fukada S., Yamamoto N., Takeda S., Tsuchida K. (2010). Mesenchymal progenitors distinct from satellite cells contribute to ectopic fat cell formation in skeletal muscle. Nat. Cell Biol..

[62] Uezumi A., Ikemoto-Uezumi M., Tsuchida K. (2014). Roles of nonmyogenic mesenchymal progenitors in pathogenesis and regeneration of skeletal muscle. Front. Physiol..

[63] Uezumi A., Ito T., Morikawa D., Shimizu N., Yoneda T., Segawa M., Yamaguchi M., Ogawa R., Matev M.M., Miyagoe-Suzuki Y. (2011). Fibrosis and adipogenesis originate from a common mesenchymal progenitor in skeletal muscle. J. Cell Sci..

[64] Heredia J.E., Mukundan L., Chen F.M., Mueller A.A., Deo R.C., Locksley R.M., Rando T.A., Chawla A. (2013). Type 2 innate signals stimulate fibro/adipogenic progenitors to facilitate muscle regeneration. Cell.

[65] Owens F.N., Gill D.R., Secrist D.S., Coleman S.W. (1995). Review of some aspects of growth and development of feedlot cattle. J. Anim. Sci..

[66] Pethick D.W., Harper G.S., Oddy V.H. (2004). Growth, development and nutritional manipulation of marbling in cattle: A review. Aust. J. Exp. Agric..

[67] Rollin X., Medale F., Gutieres S., Blanc D., Kaushik S.J. (2003). Short- and long-term nutritional modulation of acetyl-coa carboxylase activity in selected tissues of rainbow trout (oncorhynchus mykiss). Br. J. Nutr..

[68] Shingfield K.J., Bernard L., Leroux C., Chilliard Y. (2010). Role of trans fatty acids in the nutritional regulation of mammary lipogenesis in ruminants. Animal.

[69] Fujino T., Kondo J., Ishikawa M., Morikawa K., Yamamoto T.T. (2001). Acetyl-coa synthetase 2, a mitochondrial matrix enzyme involved in the oxidation of acetate. J. Biol. Chem..

[70] Vernon R.G. (1980). Lipid metabolism in the adipose tissue of ruminant animals. Progress Lipid Res..

[71] Griffin M.J., Sul H.S. (2004). Insulin regulation of fatty acid synthase gene transcription: Roles of usf and SREBP-1C. IUBMB Life.

[72] Nelson D.L., Cox M.M. (2004). Lehninger-Principles of Biochemistry.

[73] Ward R.E., Woodward B., Otter N., Doran O. (2010). Relationship between the expression of key lipogenic enzymes, fatty acid composition, and intramuscular fat content of limousin and aberdeen angus cattle. Livest. Sci..

[74] Underwood K.R., Tong J., Zhu M.J., Shen Q.W., Means W.J., Ford S.P., Paisley S.I., Hess B.W., Du M. (2007). Relationship between kinase phosphorylation, muscle fiber typing, and glycogen accumulation in longissimus muscle of beef cattle with high and low intramuscular fat. J. Agric. Food Chem..

[75] Smith S., Witkowski A., Joshi A.K. (2003). Structural and functional organization of the animal fatty acid synthase. Prog. Lipid Res..

[76] Potapova I.A., El-Maghrabi M.R., Doronin S.V., Benjamin W.B. (2000). Phosphorylation of recombinant human atp: Citrate lyase by camp-dependent protein kinase abolishes homotropic allosteric regulation of the enzyme by citrate and increases the enzyme activity. Allosteric activation of ATP: Citrate lyase by phosphorylated sugars. Biochemistry.

[77] Holness M.J., Sugden M.C. (2003). Regulation of pyruvate dehydrogenase complex activity by reversible phosphorylation. Biochem. Soc. Trans..

[78] Brownsey R.W., Boone A.N., Elliott J.E., Kulpa J.E., Lee W.M. (2006). Regulation of acetyl-coa carboxylase. Biochem. Soc. Trans..

[79] Baldwin R.L., McLeod K.R., Baumann R.G., Connor E.E. (2006). Influence of carbohydrate infusion on lipogenic enzyme and regulatory protein gene expression in growing beef steers. FASEB J..

[80] Girard J., Ferre P., Foufelle F. (1997). Mechanisms by which carbohydrates regulate expression of genes for glycolytic and lipogenic enzymes. Annu. Rev. Nutr..

[81] Matsuishi M., Fujimori M., Okitani A. (2001). Wagyu beef aroma in wagyu (Japanese black cattle) beef preferred by the Japanese over imported beef. Anim. Sci. J..

[82] Duckett S.K., Pratt S.L., Pavan E. (2009). Corn oil or corn grain supplementation to steers grazing endophyte-free tall fescue. Ii. Effects on subcutaneous fatty acid content and lipogenic gene expression. J. Anim. Sci..

[83] Graugnard D.E., Piantoni P., Bionaz M., Berger L.L., Faulkner D.B., Loor J.J. (2009). Adipogenic and energy metabolism gene networks in longissimus lumborum during rapid post-weaning growth in angus and angus x simmental cattle fed high-starch or low-starch diets. BMC Genom..

[84] Waters S.M., Kelly J.P., O'Boyle P., Moloney A.P., Kenny D.A. (2009). Effect of level and duration of dietary *n*-3 polyunsaturated fatty acid supplementation on the transcriptional regulation of delta(9)-desaturase in muscle of beef cattle. J. Anim. Sci..

[85] Oliveira D.M., Chalfun-Junior A., Chizzotti M.L., Barreto H.G., Coelho T.C., Paiva L.V., Coelho C.P., Teixeira P.D., Schoonmaker J.P., Ladeira M.M. (2014). Expression of genes involved in lipid metabolism in the muscle of beef cattle fed soybean or rumen-protected fat, with or without monensin supplementation. J. Anim. Sci..

[86] Zhang H.B., Zhang X.F., Wang Z.S., Dong X.W., Tan C., Zou H.W., Peng Q.H., Xue B., Wang L.Z., Dong G.Z. (2015). Effects of dietary energy level on lipid metabolism-related gene expression in subcutaneous adipose tissue of yellow breed x simmental cattle. Anim. Sci. J..

[87] Chilliard Y., Ferlay A., Mansbridge R.M., Doreau M. (2000). Ruminant milk fat plasticity: Nutritional control of saturated, polyunsaturated, trans and conjugated fatty acids. Ann. Zootech..

[88] Cook R.M., Miller L.D. (1965). Utilization of volatile fatty acids in ruminants. I. Removal of them from portal blood by the liver. J. Dairy Sci..

[89] Schoonmaker J.P. Effects of Lifetime Nutrition on Beef Quality. Proceedings of the III International Symposium of Beef Cattle.

[90] Du M., Zhao J.X., Yan X., Huang Y., Nicodemus L.V., Yue W., McCormick R.J., Zhu M.J. (2011). Fetal muscle development, mesenchymal multipotent cell differentiation, and associated signaling pathways. J. Anim. Sci..

[91] Hocquette J.F., Graulet B., Olivecrona T. (1998). Lipoprotein lipase activity and mRNA levels in bovine tissues. Comp. Biochem. Physiol. B.

[92] Hocquette J.F., Cassar-Malek I., Scalbert A., Guillou F. (2009). Contribution of genomics to the understanding of physiological functions. J. Physiol. Pharmacol..

[93] Smith S.B., Crouse J.D. (1984). Relative contributions of acetate, lactate and glucose to lipogenesis in bovine intramuscular and subcutaneous adipose-tissue. J. Nutr..

[94] Gilbert C.D., Lunt D.K., Miller R.K., Smith S.B. (2003). Carcass, sensory, and adipose tissue traits of brangus steers fed casein-formaldehyde-protected starch and/or canola lipid. J. Anim. Sci..

[95] Chung K.Y., Lunt D.K., Kawachi H., Yano H., Smith S.B. (2007). Lipogenesis and stearoyl-coa desaturase gene expression and enzyme activity in adipose tissue of short- and long-fed angus and wagyu steers fed corn- or hay-based diets. J. Anim. Sci..

[96] Rhoades R.D., Sawyer J.E., Chung K.Y., Schell M.L., Lunt D.K., Smith S.B. (2007). Effect of dietary energy source on *in vitro* substrate utilization and insulin sensitivity of muscle and adipose tissues of angus and wagyu steers. J. Anim. Sci..

[97] Tardif A., Julien N., Pelletier A., Thibault G., Srivastava A.K., Chiasson J.L., Coderre L. (2001). Chronic exposure to β-hydroxybutyrate impairs insulin action in primary cultures of adult cardiomyocytes. Am. J. Physiol. Endocrinol. Med..

[98] Choat W.T., Krehbiel C.R., Duff G.C., Kirksey R.E., Lauriault L.M., Rivera J.D., Capitan B.M., Walker D.A., Donart G.B., Goad C.L. (2003). Influence of grazing dormant native range or winter wheat pasture on subsequent finishing cattle performance, carcass characteristics, and ruminal metabolism. J. Anim. Sci..

[99] Schoonmaker J.P., Cecava M.J., Faulkner D.B., Fluharty F.L., Zerby H.N., Loerch S.C. (2003). Effect of source of energy and rate of growth on performance, carcass characteristics, ruminal fermentation, and serum glucose and insulin of early-weaned steers. J. Anim. Sci..

[100] Carvalho J.R., Chizzotti M.L., Ramos E.M., Machado Neto O.R., Lanna D.P., Lopes L.S., Teixeira P.D., Ladeira M.M. (2014). Qualitative characteristics of meat from young bulls fed different levels of crude glycerin. Meat Sci..

[101] Swanson K.C., Matthews J.C., Matthews A.D., Howell J.A., Richards C.J., Harmon D.L. (2000). Dietary carbohydrate source and energy intake influence the expression of pancreatic α-amylase in lambs. J. Nutr..

[102] Swanson K.C., Matthews J.C., Woods C.A., Harmon D.L. (2002). Postruminal administration of partially hydrolyzed starch and casein influences pancreatic α-amylase expression in calves. J. Nutr..

[103] Kellett G.L., Brot-Laroche E., Mace O.J., Leturque A. (2008). Sugar absorption in the intestine: The role of glut2. Annu. Rev. Nutr..

[104] Ferraris R.P., Diamond J. (1997). Regulation of intestinal sugar transport. Physiol. Rev..

[105] Liao S.F., Harmon D.L., Vanzant E.S., McLeod K.R., Boling J.A., Matthews J.C. (2010). The small intestinal epithelia of beef steers differentially express sugar transporter messenger ribonucleic acid in response to abomasal *versus* ruminal infusion of starch hydrolysate. J. Anim. Sci..

[106] Guimaraes K.C., Hazelton S.R., Matthews J.C., Swanson K.C., Harmon D.L., Branco A.F. (2007). Influence of starch and casein administered postruminally on small intestinal sodium-glucose cotransport activity and expression. Braz. Arch. Biol. Technol..

[107] Rodriguez S.M., Guimaraes K.C., Matthews J.C., McLeod K.R., Baldwin R.L., Harmon D.L. (2004). Influence of abomasal carbohydrates on small intestinal sodium-dependent glucose cotransporter activity and abundance in steers. J. Anim. Sci..

[108] Dervishi E., Serrano C., Joy M., Serrano M., Rodellar C., Calvo J.H. (2010). Effect of the feeding system on the fatty acid composition, expression of the delta9-desaturase, peroxisome proliferator-activated receptor alpha, gamma, and sterol regulatory element binding protein 1 genes in the semitendinous muscle of light lambs of the rasa aragonesa breed. BMC Vet. Res..

[109] Herdmann A., Nuernberg K., Martin J., Nuernberg G., Doran O. (2010). Effect of dietary fatty acids on expression of lipogenic enzymes and fatty acid profile in tissues of bulls. Animal.

[110] Lee J.H., Yamamoto I., Jeong J.S., Nade T., Arai T., Kimura N. (2011). Relationship between adipose maturity and fatty acid composition in various adipose tissues of Japanese black, holstein and crossbred (f1) steers. Anim. Sci. J..

[111] Bionaz M., Chen S., Khan M.J., Loor J.J. (2013). Functional role of PPARs in ruminants: Potential targets for fine-tuning metabolism during growth and lactation. PPAR Res..

[112] da Costa A.S., Pires V.M., Fontes C.M., Mestre Prates J.A. (2013). Expression of genes controlling fat deposition in two genetically diverse beef cattle breeds fed high or low silage diets. BMC Vet. Res..

[113] Aranda A., Pascual A. (2001). Nuclear hormone receptors and gene expression. Physiol. Rev..

[114] Mangelsdorf D.J., Thummel C., Beato M., Herrlich P., Schutz G., Umesono K., Blumberg B., Kastner P., Mark M., Chambon P. (1995). The nuclear receptor superfamily: The second decade. Cell.

[115] Lemay D.G., Hwang D.H. (2006). Genome-wide identification of peroxisome proliferator response elements using integrated computational genomics. J. Lipid Res..

[116] Poulsen L.L., Siersbk M., Mandrup S. (2012). PPARs: Fatty acid sensors controlling metabolism. Semin. Cell Dev. Biol..

[117] Brun R.P., Tontonoz P., Forman B.M., Ellis R., Chen J., Evans R.M., Spiegelman B.M. (1996). Differential activation of adipogenesis by multiple PPAR isoforms. Genes Dev..

[118] Desvergne B., Wahli W. (1999). Peroxisome proliferator-activated receptors: Nuclear control of metabolism. Endocr. Rev..

[119] Kersten S. (2014). Integrated physiology and systems biology of PPARα. Mol. Metab..

[120] Olefsky J.M., Saltiel A.R. (2000). PPARγ and the treatment of insulin resistance. Trends Endocrinol. Metab..

[121] Bunger M., van den Bosch H.M., van der Meijde J., Kersten S., Hooiveld G.J., Muller M. (2007). Genome-wide analysis of PPARα activation in murine small intestine. Physiol. Genom..

[122] Tyagi S., Gupta P., Saini A.S., Kaushal C., Sharma S. (2011). The peroxisome proliferator-activated receptor: A family of nuclear receptors role in various diseases. J. Adv. Pharm. Technol. Res..

[123] Bionaz M., Thering B.J., Loor J.J. (2012). Fine metabolic regulation in ruminants via nutrient–gene interactions: Saturated long-chain fatty acids increase expression of genes involved in lipid metabolism and immune response partly through PPAR-α activation. Br. J. Nutr..

[124] Lim S., Jang H.J., Park E.H., Kim J.K., Kim J.M., Kim E.K., Yea K., Kim Y.H., Lee-Kwon W., Ryu S.H. (2012). Wedelolactone inhibits adipogenesis through the ERK pathway in human adipose tissue-derived mesenchymal stem cells. J. Cell. Biochem..

[125] Varga T., Nagy L. (2008). Nuclear receptors, transcription factors linking lipid metabolism and immunity: The case of peroxisome proliferator-activated receptor γ. Eur. J. Clin. Investig..

[126] Sharma S., Sun X.T., Rafikov R., Kumar S., Hou Y.L., Oishi P.E., Datar S.A., Raff G., Fineman J.R., Black S.M. (2012). Ppar-gamma regulates carnitine homeostasis and mitochondrial function in a lamb model of increased pulmonary blood flow. PLoS ONE.

[127] Eberle D., Hegarty B., Bossard P., Ferre P., Foufelle F. (2004). SREBP transcription factors: Master regulators of lipid homeostasis. Biochimie.

[128] Shimano H., Horton J.D., Shimomura I., Hammer R.E., Brown M.S., Goldstein J.L. (1997). Isoform 1c of sterol regulatory element binding protein is less active than isoform 1a in livers of transgenic mice and in cultured cells. J. Clin. Investig..

[129] Tontonoz P., Kim J.B., Graves R.A., Spiegelman B.M. (1993). ADD1-A novel helix-loop-helix transcription factor associated with adipocyte determination and differentiation. Mol. Cell. Biol..

[130] Desvergne B., Michalik L., Wahli W. (2006). Transcriptional regulation of metabolism. Physiol. Rev..

[131] Obsen T., Faergeman N.J., Chung S., Martinez K., Gobern S., Loreau O., Wabitsch M., Mandrup S., McIntosh M. (2012). *Trans*-10, *cis*-12 conjugated linoleic acid decreases *de novo* lipid synthesis in human adipocytes. J. Nutr. Biochem..

[132] Botolin D., Wang Y., Christian B., Jump D.B. (2006). Docosahexaneoic acid (22:6,*n*-3) regulates rat hepatocyte srebp-1 nuclear abundance by ERK- and 26s proteasome-dependent pathways. J. Lipid Res..

[133] Tsuboyama-Kasaoka N., Takahashi M., Tanemura K., Kim H.J., Tange T., Okuyama H., Kasai M., Ikemoto S., Ezaki O. (2000). Conjugated linoleic acid supplementation reduces adipose tissue by apoptosis and develops lipodystrophy in mice. Diabetes.

[134] Oswal D.P., Balanarasimha M., Loyer J.K., Bedi S., Soman F.L., Rider S.D., Hostetler H.A. (2013). Divergence between human and murine peroxisome proliferator-activated receptor α ligand specificities. J. Lipid Res..

[135] Teixeira P.D. (2015). A Subespécie e a Dieta Afetam a Expressão de Genes Envolvidos no Metabolismo Lipídico e a Composição Química do Musculo de Bovino de Corte (the Subspecies and Diet Affetcs the Expression of Genes Involved in Lipid Metabolism and the Chemical Composition of Skeletal Muscle in Beef Cattle). http://repositorio.ufla.br/handle/1/5566.

[136] Brown J.M., Boysen M.S., Jensen S.S., Morrison R.F., Storkson J., Lea-Currie R., Pariza M., Mandrup S., McIntosh M.K. (2003). Isomer-specific regulation of metabolism and PPARγ signaling by CLA in human preadipocytes. J. Lipid Res..

[137] Sanosaka M., Minashima T., Suzuki K., Watanabe K., Ohwada S., Hagino A., Rose M.T., Yamaguchi T., Aso H. (2008). A combination of octanoate and oleate promotes *in vitro* differentiation of porcine intramuscular adipocytes. Comp. Biochem. Phys. B.

[138] Smith S.B., Kawachi H., Choi C.B., Choi C.W., Wu G., Sawyer J.E. (2009). Cellular regulation of bovine intramuscular adipose tissue development and composition. J. Anim. Sci..

[139] Jenkins T.C., Harvatine K.J. (2014). Lipid feeding and milk fat depression. Vet. Clin. N. Am. Food Anim. Pract..

[140] Brandebourg T.D., Hu C.Y. (2005). Isomer-specific regulation of differentiating pig preadipocytes by conjugated linoleic acids. J. Anim. Sci..

[141] Jurie C., Cassar-Malek I., Bonnet M., Leroux C., Bauchart D., Boulesteix P., Pethick D.W., Hocquette J.F. (2007). Adipocyte fatty acid-binding protein and mitochondrial enzyme activities in muscles as relevant indicators of marbling in cattle. J. Anim. Sci..

[142] Peng Q.H., Wang Z.S., Tan C., Zhang H.B., Hu Y.N., Zou H.W. (2012). Effects of different pomace and pulp dietary energy density on growth performance and intramuscular fat deposition relating mRNA expression in beef cattle. J. Food Agric. Environ..

[143] Joseph S.J., Robbins K.R., Pavan E., Pratt S.L., Duckett S.K., Rekaya R. (2010). Effect of diet supplementation on the expression of bovine genes associated with fatty acid synthesis and metabolism. Bioinform. Biol. Insights.

[144] Waylan A.T., Dunn J.D., Johnson B.J., Kayser J.P., Sissom E.K. (2004). Effect of flax supplementation and growth promotants on lipoprotein lipase and glycogenin messenger rna concentrations in finishing cattle. J. Anim. Sci..

[145] Hausman G.J., Barb C.R., Dean R.G. (2008). Patterns of gene expression in pig adipose tissue: Insulin-like growth factor system proteins, neuropeptide Y (NPY), NPY receptors, neurotrophic factors and other secreted factors. Domest. Anim. Endocrinol..

[146] Shin J., Li B., Davis M.E., Suh Y., Lee K. (2009). Comparative analysis of fatty acid-binding protein 4 promoters: Conservation of peroxisome proliferator-activated receptor binding sites. J. Anim. Sci..

[147] Kim K.H. (1997). Regulation of mammalian acetyl-coenzyme a carboxylase. Annu. Rev. Nutr..

[148] Abu-Elheiga L., Matzuk M.M., Abo-Hashema K.A.H., Wakil S.J. (2001). Continuous fatty acid oxidation and reduced fat storage in mice lacking acetyl-coa carboxylase 2. Science.

[149] Alvarez M.J., Diez A., Lopez-Bote C., Gallego M., Bautista J.M. (2000). Short-term modulation of lipogenesis by macronutrients in rainbow trout (oncorhynchus mykiss) hepatocytes. Br. J. Nutr..

[150] Ladeira M.M., Oliveira D.M., Chalfun Júnior A., Chizzotti M.L., Barreto H.G., Coelho T.C. (2013). Gene expression of lipogenic enzymes present in muscle of bullocks fed ground soybean grain or ground cottonseed and vitamin E. J. Anim. Sci..

[151] Flowers M.T., Ntambi J.M. (2008). Role of stearoyl-coenzyme a desaturase in regulating lipid metabolism. Curr. Opin. Lipidol..

[152] Yang A., Larsen T.W., Smith S.B., Tume R.K. (1999). Delta(9) desaturase activity in bovine subcutaneous adipose tissue of different fatty acid composition. Lipids.

[153] Kadegowda A.K.G., Burns T.A., Pratt S.L., Duckett S.K. (2013). Inhibition of stearoyl-coa desaturase 1 reduces lipogenesis in primary bovine adipocytes. Lipids.

[154] Zhang H.B., Wang Z.S., Peng Q.H., Tan C., Zou H.W. (2014). Effects of different levels of protein supplementary diet on gene expressions related to intramuscular deposition in early-weaned yaks. Anim. Sci. J..

[155] Renaville B., Mullen A., Moloney F., Larondelle Y., Schneider Y.J., Roche H.M. (2006). Eicosapentaenoic acid and 3,10 dithia stearic acid inhibit the desaturation of *trans*-vaccenic acid into *cis*-9, *trans*-11-conjugated linoleic acid through different pathways in caco-2 and t84 cells. Br. J. Nutr..

[156] Sampath H., Ntambi J.M. (2006). Stearoyl-coenzyme a desaturase 1, sterol regulatory element binding protein-1c and peroxisome proliferator-activated receptor-α: Independent and interactive roles in the regulation of lipid metabolism. Curr. Opin. Clin. Nutr..

[157] Schmitz G., Ecker J. (2008). The opposing effects of *n*-3 and *n*-6 fatty acids. Prog. Lipid Res..

